# Assent, parental consent and reconsent for health research in Africa: thematic analysis of national guidelines and lessons from the SickleInAfrica registry

**DOI:** 10.1186/s12910-022-00843-3

**Published:** 2022-12-08

**Authors:** Nchangwi Syntia Munung, Victoria Nembaware, Lawrence Osei-Tutu, Marsha Treadwell, Okocha Emmanuel Chide, Daima Bukini, Hilda Tutuba, SickleInAfrica ELSI WG, Malula Nkanyemka, Kofi Anie, Charmaine Royale, Ambroise Wonkam

**Affiliations:** 1 Division of Human Genetics, University of Cape Town, Cape Town, South Africa; 2 Komfe-Anokye Teaching Hospital, Kumasi, Ghana; 3 Department of Pediatrics, School of Medicine, University of California San Francisco, San Francisco, CA USA; 4 Nnamdi Azikiwe University Teaching Hospital, Nnewi, Anambra Nigeria; 5 Sickle Cell Program, Muhimbili University of Health and Allied Sciences, Dar Es Salaam, Tanzania; 6 McKusick-Nathans Institute and Department of Genetic Medicine, Johns Hopkins Medicine, Baltimore, MD USA

**Keywords:** Assent, Parental consent, Reconsent, Disease registries, Minors

## Abstract

The enrolment of children and adolescents in health research requires that attention to be paid to specific assent and consent requirements such as the age range for seeking assent; conditions for parental consent (and waivers); the age group required to provide written assent; content of assent forms; if separate assent and parental consent forms should be used, consent from emancipated young adults; reconsent at the age of adulthood when a waiver of assent requirements may be appropriate and the conditions for waiving assent requirements. There is however very little available information for researchers and ethics committees on how to navigate these different issues. To provide guidance to research initiatives, the SickleInAfrica consortium conducted a thematic analysis of a sample of research ethics guidelines and procedures in African countries, to identify guidance for assent requirements in health research. The thematic analysis revealed that 12 of 24 African countries specified the age group for which assent is required. The minimum age for written assent varied across the countries. Five countries, Algeria, Botswana, Cameroon, Nigeria and The Democratic Republic of Congo require consent from both parents/family council in certain circumstances. Botswana, Nigeria, South Africa and Uganda have specific assent/consent requirements for research with emancipated minors. South Africa and Algeria requires re-consent at onset of adulthood. Five countries (Botswana, Cameroon, Nigeria, South Africa and Tanzania) specified conditions for waiving assent requirements. The CIOMS and the ICH-GCP guidelines had the most comprehensive information on assent requirements compared to other international guidelines. An interactive map with assent requirements for different African countries is provided. The results show a major gap in national regulations for the inclusion of minors in health research. The SickleInAfrica experience in setting up a multi-country SCD registry in Africa highlights the need for developing and harmonising national and international guidelines on assent and consent requirements for research involving minors. Harmonisation of assent requirements will help facilitate collaborative research across countries.

## Introduction

The inclusion of minors (children and adolescents) in registries and biobanks has several benefits for healthcare and research especially for early onset genetic conditions such as sickle cell disease (SCD). However, the inclusion of minors in research raises specific ethical issues around assent and assent comprehension [1, 2]; the age or developmental stage at which assent may be appropriate [3, 4]; parental consent [5], consent from emancipated young adults [6], reconsent at the age of adulthood [7, 8] and the importance of taking into consideration socio-cultural factors that may impact assent and parental consent [9, 10].

With growing interest in secondary uses of health data for research, there has been significant scholarship on informed consent guidelines to support biobanking, data sharing and the use of health data for secondary analysis. However, this has not been matched by a corresponding interest in highlighting national requirements for the inclusion of minors in research, including the potential impact of national research regulation on cross country collaborations, and the governance of health registries that enrol minors. Similarly, as biomedical research becomes reliant on biobanks and databases, there are questions on whether re-consent is required when minors reach the legal age of adulthood while a study is still in progress. [7]. The reason being that once a minor becomes an adult, it is necessary to ascertain that their continuous participation in a research project reflects their own choices rather than that of their parents or legal guardians [11]. This is in line with the research ethics principle of autonomy.

Very little has been written on paediatric research ethics in Africa and it is unclear if African countries have guidelines for involving children in health research [12]. In our own experience with setting up a multi-country SCD registry in Africa [13], the absence of clear national guidelines on assent and parental consent has been a major challenge in deciding how to design processes and forms for obtaining assent and parental/proxy consent. When available, the assent and consent guidelines differed from one country to another, making the creation of a harmonised multinational African SCD registry challenging. Also, because most of the participants enrolled into the SickleInAfrica registry are minors, re-consent at adulthood is emerging as an ethical issue that the consortium may have to consider given that it is collecting longitudinal data. To provide guidance to research initiatives that involve minors as participants, SickleInAfrica examined the current research ethics framework in African countries to extract specific national requirements for assent and consent in health research involving minors.

## Methodology

We undertook a thematic analysis [14] of research ethics guidelines in African countries to identify national requirements for assent, parental consent and re-consent in health research. For each guideline or standard operating procedure (SOPs) of the local REC, we extracted information on required age of assent, type of assent (written or verbal), procedures, waivers, conditions for parental consent and requirements and procedures for re-consent.

Considering that many of the national guidelines and SOPs referred to international frameworks such as the Council for International Organizations of Medical Sciences (CIOMS) guidelines [15], the Declaration of Helsinki [16], the United Nations Educational, Scientific and Cultural Organization Universal Declaration on Bioethics and Human Rights [17], and the International Council for Harmonisation’s Guideline for Good Clinical Practice [18], we also checked for specific information on assent, parental consent and re-consent in those guidelines. This was based on the assumption that in the absence of specific national guidelines on an ethical issue, researchers and research ethics committees will refer to the recommended international guideline (s) Also, because SickleInAfrica is funded by the U.S. National Institutes of Health (NIH), we reviewed the NIH Policy and Guidelines on the *Inclusion of Individuals Across the Lifespan as Participants in Research Involving Human Subjects* (https://grants.nih.gov/policy/inclusion/lifespan.htm], as well as the U.S. Department of Health and Human Services (HHS) Office for Human Research Protections International Program [https://www.hhs.gov/ohrp/international/).

We conclude with a brief description of how the SickleInAfrica consortium [13], has proceeded with requirements for assent and parental consent in the absence of specific national requirements for assent and parental consent. SickleInAfrica currently enrols both minors and adults with SCD. Phase one of the project included enrolment sites in Ghana, Nigeria and Tanzania.

### Search for African national ethics guidelines

We sourced for national research ethics guidelines using a variety of approaches, including a repertoire of national ethics guidelines from a previous study on African national regulations for biobanking [19], the ClinRegs database [20]; and the World Health Organisation’s database for National Ethics Committees (https://apps.who.int/ethics/nationalcommittees/). We also conducted a google search using the syntax: “Name of country AND research ethics guidelines” for all African countries. International research ethics guidelines frequently cited as reference documents in standard operating procedures and guidelines for RECs in Africa, namely, The Declaration of Helsinki [16], the CIOMs guidelines[15] and the ICH-GCP, were also analysed for information on assent requirements.

### Data synthesis

All guidelines were imported into NVivo 12, a qualitative data analysis software [21], for deductive thematic analysis [22, 23]. The deductive coding scheme covered: age of written assent, conditions for assent, requirements for parental consent; conditions of waiver of assent and reconsent at adulthood. NM did the initial coding of the national guidelines. The information for each country was then cross-checked by VN. As there were no discrepancies in the thematic coding, a third reviewer was not needed.

## Results

**Fig. 1:**
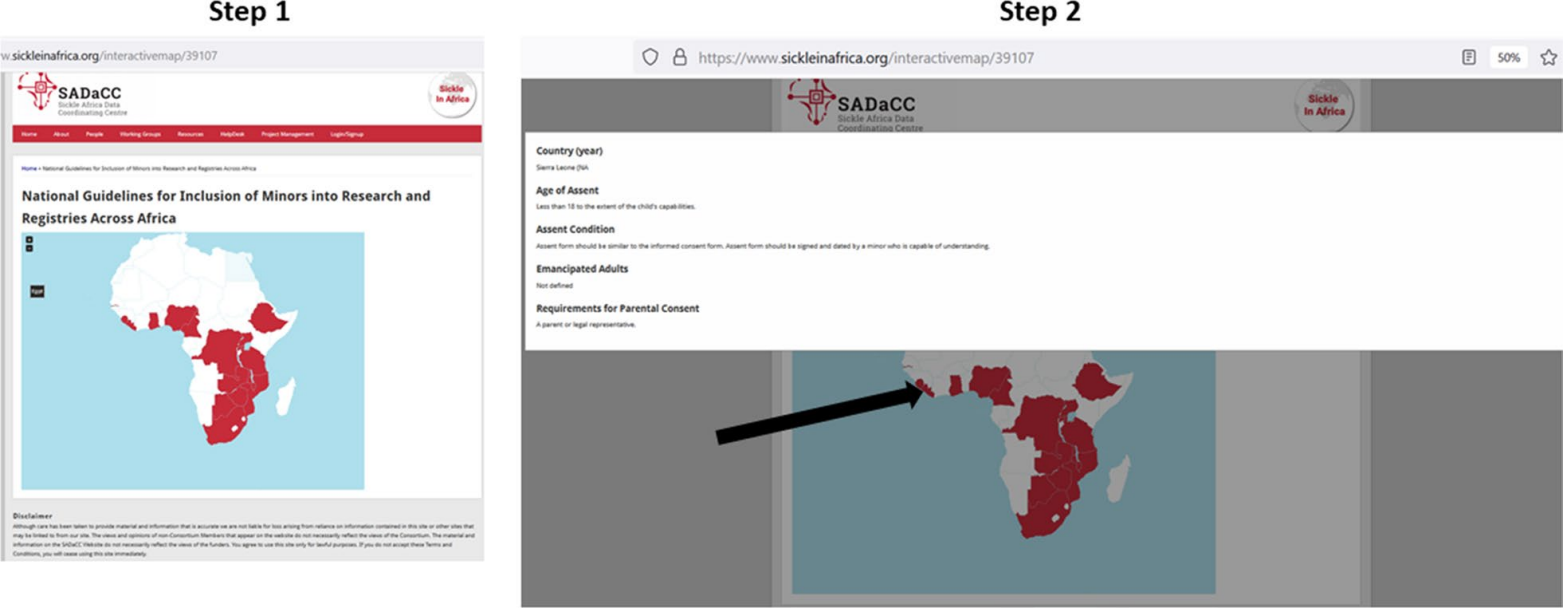
Screenshot of interactive map showing specific requirements per country (https://www.sickleinafrica.org/interactivemap/39107)

We retrieved national guidelines for just 24 African countries (Fig. 1). For some of the remaining countries, we cannot confirm whether such guidelines exist. However, previous studies have reported the absence of health research ethics guidelines in many African countries [19, 24]. An online interactive map (https://www.sickleinafrica.org/interactivemap/39107) was developed to provide stakeholders with summaries of country-specific regulations for inclusion of minors in research studies based on this report. The interactive map shows the countries for which data on assent is available. By hovering on the map of a specific country, a pop-up window comes up with country-specific guidelines.

Of the national guidelines that were analysed, eighteen had information on at least one of the themes (assent requirements) in the analysis framework (Table 1). The guidelines for many African countries referred to at least one of five international human research ethics guidelines namely: the Declaration of Helsinki; the CIOMS guidelines; WHO operational guidelines for ethics committees; UNESCO Council of Bioethics and the ICH-GCP Guidelines. Therefore, in the absence of specific national requirements, researchers and RECs may have to refer to these documents for guidance. However, only the CIOMS guidelines and the ICH-GCP guidelines provide in-depth information on assent requirements (Table 2).

**Table 1:** African National Requirements for Assent, Parental Consent and Reconsent at Adulthood

Country	Year	Age of assent	Assent condition	Emancipated minors	Parental consent	Waivers for assent and parental consent
Algeria	2018	Not specified	Consent should be adopted for minors. Not stated if written or verbal assent is required	Not specified	Authorization from a single for no more than minimal risk studies. For studies that pose more than minimal risk, authorsiation is required from the familiy council	Not specified
Botswana	2012	7–17	Not stated if written or verbal but an assent template is provided	14–17 years and married or pregnant. If an emancipated minor consent to their child’s participation, a court order should be copied and included in the research records with the consent document	Both parents must provide consent unless one parent is deceased, unknown, incompetent, or not reasonably available: or a legal guardian For less than minimal risk studies, the REC may approve consent from one parent For greater than minimal risk studies, the REC may approve consent from one parent	Requirements for assent may be waived: •If the research offers the child, the possibility of a direct benefit and available only in the context of the research •When consent may be waived
Cameroon	2012	14–20	Written assent Language and syntax of the assent form should be similar to that in the parental consent form	Less than 21 and married, pregnant, mother, head of household etc	For no more than minimal risk studies, consent may be obtained from at least one parent/guardian For greater than minimal risk studies, consent should be obtained from both parents except where only one parent has legal responsibility or custody of the child	Assent may be waived: •If the intervention is of direct health benefit to child and only available in the research setting •If the study meets conditions for a waiver of consent
Democratic Republic of Congo	2011	13–17	Written assent. Researchers can decide on a higher age group for obtaining assent based on the complexity of study	Not specified	Consent from both parents or guardian	Not specified
Ethiopia	2014	12–17	Not specified if written or verbal. However, template assent form is provided	Persons less than 18 working or earning their living, married, parenting	Consent of at least one parent, next-of-kin, or guardian	Not specified
Gambia		12–17	Not specified	Not specified	Not specified	Not specified
Ghana	2015	8–17 Years	Written assent	Not specified	Not specified	Not specified
Liberia	2019	15–17	Written assent	Not specified	Not specified	Not specified
Malawi	2003	7–17 Years	Assent tailored to the level of comprehension	Legally married and Students under a defined Malawian law	Not specified	Not specified
Mozambique	2014	Above 16	Not specified	Not specified	A legal parent or guardian	Not specified
Nigeria	2016	12–17 years	Not specified	Researchers to justify in their research protocols when a child may be declared as ‘emancipated’ Emancipated minors can give consent in their cognizance	Less than 12: Consent of both parents or the parent/legal guardian that has primary responsibility for the child at the time of research Above 12- A relevant parent/legal guardian	Waiver of parental consent may be granted for: •No more than minimal risk studies •Study holds out potential to benefit the children being involved in the study •Study objectives could not otherwise be achieved if parents have to be consented
Rwanda	2009	9–20 Years	Written assent	Not specified	A parent or legal guardian	Not specified
South Africa	2015	Less than 18	For minors less than 7 years assent form should be read to them Written assent for minors 7 years and above. The older the minor, the more the assent form should mirror the parental consent	An unmarried minor parent may not consent to their child’s participation in research as pregnancy and childbirth do not change the legal status of a minor mother	A parent or legal guardian	Parental consent may be waived •For minimal risk research in particular circumstances: •For reasons of sensitivity, like discussions about sexual activities, substance abuse in minors 16 years and older •Where recruiting enough minors may be a challenge because they may be unwilling to participate if parental permission is required
Tanzania	2009	Not specified	Not specified	Not specified	The permission of one parent or guardian is sufficient if consistent with the laws of the country	RECs may waive the assent requirement if: •The study involves no more than minimal risk •The study cannot be carried out without the waiver
Sierra Leone	NA	Less than 18 to the extent of the child`s capabilities	Assent form should be similar to the informed consent form Assent form should be signed and dated by a minor who is capable of understanding	Not specified	A parent or legal representative	Not specified
Uganda	2014	8–17 years	Not specified	Mature minors are persons 14–17 years who have drug or alcohol dependency, or a sexually transmitted infection, individuals below the age of majority who are pregnant, married, have a child or cater for their own livelihood Mature and emancipated minors may independently provide informed consent as deemed necessary by REC	Parent or guardian	Not specified
Zambia	2013	Not Specified	Written assent required when the minor can understand the nature and potential risks and benefits of the study	Not specified	A parent or legal guardian	Not specified
Zimbabwe (JREC)*	2011	5–18 Years *	Less than 5 (oral) 5–12 years (written tailored to child’s cognitive level) 13–18 years (same as parental consent)	Not specified	Not specified	Not specified

^*^Joint Research Ethics Committee for University of Zimbabwe College of Health Sciences and Parirenyatwa Group Hospitals (JREC)

**Table 2:** International guidelines on the involvement of minors in health research

International Guideline	Assent	Requirements for parental consent	Re consent at age of adulthood	Emancipated Minors	Waiver of Assent/Parental Consent
Declaration of Helsinki	All vulnerable populations	Consent required from a legally authorized representative	Not specified	Not specified	Not specified
CIOMS Guidelines	Tailored to the child’s or adolescent’s level of maturity	A parent or a legally authorized representative	Re-consent when a minor reaches the legal age of maturity during the research	Not specified	A REC may waive requirements for assent/parental consent if: •The research would not be feasible or practicable to carry out without the waiver •The research has important social value, and the research poses no more than minimal risks to
UNESCO Declaration on Bioethics and Human Rights	Generic-all vulnerable populations	Not specified	Not specified	Not specified	Not specified
International Council for Harmonisation’s Guideline for Good Clinical Practice. 2017	Age of assent should be consistent with local legal requirements Minors of appropriate intellectual maturity should sign and date either the assent or informed consent form	Parental consent should be obtained in accordance with regional laws or regulation	informed consent for continued participation is required once a child reaches the age of legal consent	Emancipated or mature minors (defined by local laws) may give autonomous consent	Not specified

### Age group for which assent is required and type of assent

Twelve (12) out of the 24 countries, specified the age range for which assent is required. This varied significantly across countries, with Zimbabwe, Botswana and South Africa having the lowest age group (5–7 years) for which assent is required. Written assent is explicitly stated in the guidelines of eight countries and two countries (South Africa and Zimbabwe) had specific recommendations for written assent based on age group. For example, the South African regulation stipulates that the assent form should be read to minors less than 7 years old, and for 7–17-year-olds, the assent form should mirror the consent form as the minor gets older. In Zimbabwe, the Joint Research Ethics Committee for University of Zimbabwe College of Health Sciences and Parirenyatwa Group Hospitals (JREC) recommends verbal assent for children less than 5 years and written assent for children 5–18 years. The JREC requires that the assent form for 5–12 years old be tailored to the child’s cognitive level, while for 13–17 years, the content of the assent form should be the same as that of the parental consent form. The ICH-GCP guidelines recommend that that the age of assent should be consistent with local legal requirements and that minors of appropriate intellectual maturity should sign and date either the assent or informed consent form.

### Parental consent requirements

The specific requirements for parental consent varies across countries. Although all 24 countries required parental consent, many did not outline specific requirements. Botswana and the Democratic Republic of Congo require consent from both parents, while for Nigeria, the consent of both parents should be obtained if the child is less than 12 years, otherwise, the parent/legal guardian that has primary responsibility for the child at the time of research can provide consent. In Cameroon, the parental consent condition is based on the risk level of the study.

### Emancipated minors: assent and consent requirements

The guidelines for Botswana, Cameroon, Ethiopia, Malawi, Nigeria, South Africa and Uganda refer to emancipated minors (Table 1). However, with the exception of South Africa. In no specific recommendations are made on assent/consent for emanicipated minors. In South Africa, emancipated minors can consent for themselves but not for their child.

### Waiver of assent and parental consent requirement

Five countries (Botswana, Cameroon, Nigeria, South Africa and Tanzania) had specific guidelines on waiver of assent and parental consent (Table 1). The common scenario whereby RECs may approve a waiver of consent are: (1) minimal risk studies; (2) if the intervention under of direct health benefit to the child and only available in the research setting; (3) if the research meets conditions for a waiver of informed consent and (4) where study objectives could not otherwise be achieved if parents were to be consented. The CIOMs guidelines make similar recommendations for the waiver of assent and parental consent.

### Reconsent at the age of adulthood

The South Africa and Algeria guidelines require that when a minor turns 18 years during the study, they should be approached at the time of their birthday to enable them to give consent. However, if the study is no longer actively recruiting or interacting with research participants, re-consent at adulthood may not be required. The CIOMS and ICH-GCP guidelines recommend that informed consent for continued participation is required once a child reaches the age of legal consent.

### SickleInAfrica: navigating issues of assent, parental consent and reconsent in the absence of national guidelines or regulation

SickleInAfrica is currently enrolling participants in different African countries into a SCD registry [13]. The main purpose of the registry is to facilitate research on SCD. SickleInAfrica is an NIH funded project and is therefore expected to comply to NIH policies on human subject research. The NIH provides clear guidelines on assent, parental consent and re-consent at the age of majority. NIH policy is that no child may be enrolled, screened, or have research procedures initiated, unless parental permission and child assent has been obtained. For research taking place at an NIH site, and in cases where parents share joint legal custody for medical decision-making of a child, both parents must give their permission regardless of the risk level of the research, except in the case where one parent has since died, become incompetent, or is not reasonably available. Researchers are also expected to obtain consent for continued participation in a study if a child reaches the age of majority during the research, unless when consent is not required or has been waived by a REC. Per NIH policies, legally emancipated minors are considered adults. Human subjects research funded by the NIH must adhere to regulations of the host country, however, if regulations differ, the most restrictive one takes effect. Given that SCD is an early onset genetic disease, the majority of SickleInAfrica registry participants are minors. Therefore, SickleInAfrica had to ensure practices are in line with national requirements and the NIH policy. The challenges faced by the consortium were the following:

#### Challenge 1: Lack of a harmonized age for assent across the consortium

The first phase of SickleInAfrica involved enrolment sites in Ghana, Tanzania and Nigeria. The national requirements for assent and consent differed in these countries (Table 1). In Tanzania, for example, the national ethics guidelines do not provide specific information on assent, including the age for which assent is required and the type of assent (written or verbal). However, the legal age for adulthood as stated in the Tanzania constitution is 18 years. Nigeria, on the other hand, requires assent for minors 12–17 years, while for Ghana, written assent must be obtained for children 8–17 years. To ensure that procedures and policies for data collection were harmonised across SickleInAfrica sites, the consortium recommended that written assent, in addition to parental consent, be obtained for children 8–17 years.

#### Challenge 2: Lack of harmonized specific guidelines on parental consent

For the three SickleInAfrica countries, only Nigeria had specific guidelines for parental consent, with different requirements based on the age group (Table 1). The major logistical constraint to parental consent at the Nigeria site was obtaining consent from both parents when the child is less than 12 years. This was because, it is common for just one parent, usually the mother, to accompany a child to the hospital. To overcome this challenge, the SickleInAfrica Nigeria site obtained consent from the available parent (usually the mother) at the time of the enrolment. In Ghana, it is no specific requirement for parental consent, therefore, consent was obtained from at least one parent or guardian.

## Discussion and conclusion

Many African countries have limited guidance for assent and parental consent in health research and the guidelines vary from country to country. A similar observation has been reported for the European Union [25]. International research ethics guidelines, except for the CIOMS and ICH-GCP guidelines, also have very minimal guidance on assent and parental consent. The SickleInAfrica [13] experience demonstrates how this could pose a challenge for cross-country African collaborations that seek to involve paediatric and adolescent populations in health research. A scoping review on HIV adolescent research in LMICs has also highlighted how the absent of guidelines on assent and parental consent could pose a major challenge for health research involving older minors [12].

Currently, only South Africa and Algeria have guidelines for re-consent at the legal age of adulthood. The CIOMS and the ICH-GCP guidelines recommend re-consent when a minor reaches the legal age of adulthood. Considering that many African national guidelines refer to the CIOMS guidelines, initiatives seeking to involve minors in research should consider developing procedures for re-consent at age of adulthood. However, there are concerns that re-consenting may not be feasible or cost effective [26]. To overcome this challenge, alternatives to re-consent, such as notification-only or opt-out phone or email messages may be considered.

Disease registries that collect clinical records with the goal of using the de-identified data for research purposes, pose no more than minimal risk to participants. It is worth exploring if research ethics committees may grant a waiver of active parental consent for older minors that are able to read and comprehend research information presented in consent documents search procedures [29–31]. This has the advantage of reducing logistical challenges of obtaining parental consent for minimal risk studies. Empirical studies have shown that minors above the age of eleven tend to have a greater understanding of research procedures [27, 28]. Empirical research across African countries to explore understanding of information provided during assent will be critical in determining cognitive attributes necessary to obtain valid assent for different age groups; and if waiver of parental consent could an option for minimal risk studies involving older minors. It will also be important to identify other gatekeeping ethics other procedures or activities that should be done in the case where parental consent is waived. The South African guidelines for example, recommend prior engagement with participating community role players for studies that have had REC approval to obtain independent consent from minors.

Overall, there is a need to revisit the bioethics discourse on assent and parental consent for minimal risk health research and to revise and harmonise existing guidance on consent requirements for research involving minors. Our analysis, which focussed mainly on written guidelines, revealed gaps in both national and international research regulation involving minors in health research registry research. We however note that written guidelines, or the absence thereof, do not often reflect the actual decisions made by RECs, as RECs may have specific requirements that speak to ethical issues not be covered in national guidelines or for which there is a lack of procedural clarity.

A limitation of this study is the analyses of guidelines for only 24 of 56 African countries. It is likely we may have missed the guidelines for some countries especially if the guidelines are not publicly available. Worthy of note is that some African countries do not yet have national guidelines on health research ethics. The interactive map that we have developed is an expandable resource where more guidelines could be added in the future.

## Data Availability

Data sharing is not applicable to this article as no datasets were generated during the current study. Access to some of the national guidelines can be accessed by searching the ClinRegs database (https://clinregs.niaid.nih.gov/about.php); the World Health Organisation’s database for National Ethics Committees (https://apps.who.int/ethics/nationalcommittees/nec.aspx); or by contacting Nchangwi S Munung (nchangwisyntia@yahoo.com).
